# Variation in species diversity of deep-water megafauna assemblages in the Caribbean across depth and ecoregions

**DOI:** 10.1371/journal.pone.0201269

**Published:** 2018-08-01

**Authors:** Iván Hernández-Ávila, Edlin Guerra-Castro, Carolina Bracho, Martin Rada, Frank A. Ocaña, Daniel Pech

**Affiliations:** 1 Laboratorio de Biodiversidad Marina y Cambio Climático, El Colegio de la Frontera Sur, Campeche, Mexico; 2 Consejo Nacional de Ciencia y Tecnología, Unidad Multidisciplinaria de Docencia e Investigación Sisal, Facultad de Ciencias, Universidad Nacional Autónoma de México, Puerto de Abrigo, Sisal, Yucatán, Mexico; 3 Escuela de Ciencias Aplicadas del Mar, Universidad de Oriente, Margarita Island, Venezuela; 4 Departamento de Ciencias, Universidad de Oriente, Margarita Island, Venezuela; Naturhistoriska riksmuseet, SWEDEN

## Abstract

Diversity patterns of the deep-sea megafauna in the Caribbean Basin and the Guiana ecoregion were analyzed in order to test the hypothesis of species richness variation as a function of depth and the hypothesis of non-differences between ecoregions by analyzing spatial patterns of five taxa and a merged assemblage. Collections of five taxa (corals, sea stars, sea urchins, sea lilies and gastropods) were obtained from seven oceanographic expeditions aboard the R/V Pillsbury at 310 stations between 60 and 7500 m depth. Data were sorted according to depth zones and ecoregions and were analyzed in order to estimate species richness, changes in species composition and distinction of β-diversity by species turnover or by nestedness. The observed patterns of diversity were consistent between taxa and their assemblage: Species richness increased from the continental shelf (60–200 m deep) to the slope (200–2000 m deep), followed by a decrease at the continental rise-abyssal zone. We detected marked changes in species composition according to depth ranges. Changes in species composition in relation to ecoregions were also detected. In general, the Caribbean Basin lacks important physical barriers, causing high deep-sea ecosystem connectivity; however, variation in composition could be related to changes in environmental conditions associated with productivity and/or continental influences.

## Introduction

The Caribbean Sea is an important hotspot of marine shallow and deep-water diversity in the Atlantic Ocean [1, 2]. The diversity of deep-water megafauna such as corals [3], echinoderms [4] and other taxa [5] in the area tends to be higher than in other Atlantic provinces. This semi-enclosed sea of 2.75x10^6^ km^2^ has an average depth of 2400 m with approximately 6% of the area corresponding to shallow-water and shelf depth (< 200 m). The presence of ridges, sills and trenches separates the Caribbean seafloor into the Grenadian, Venezuelan, Colombian and Yucatan basins and the Cayman Trough. The Greater and Lesser Antilles form the Northern and Eastern boundaries with the Atlantic Ocean, the Gulf of Mexico and the Bahamas-Florida regions. The habitats of the Caribbean deep-sea benthos include hydrothermal vents, methane seep, volcanoes, mud volcanoes, seamounts, ridges and deep-waters reefs. Moreover, the basin exhibits different environmental configurations of water masses [6–8] and particulate organic carbon influx [8, 9]. Such spatial heterogeneity allows for a high spatial β-diversity of benthic organisms, both vertically (i.e., across depth) and horizontally (i.e., across regions). This, however, has not been tested.

Assessing diversity patterns of deep-sea benthic communities in the Caribbean Basin represents a major challenge due to the limited logistical capacity of Caribbean countries for deep-sea exploration and the intrinsic challenges involved in studying these ecosystems [5]. However, there are some good examples of recent approaches to conducting an exhaustive exploration of the Colombian continental margin [10] and the analysis of deep-water coral assemblages in Colombia, Curaçao and Honduras [11–14]. Benthic assessments have been extended to investigate the fauna in the sill passages of the Greater Antilles [15], cold seeps in the Barbados Prism, Trinidad and Tobago, and Colombia [16–19], hydrothermal vents of Cayman Trough and Grenada [20–22] and the deep-water effects of eruptions on Montserrat Island [23]. However, despite past and current efforts, many gaps concerning the pattern of diversity across depths and different ecoregions in the Caribbean Basin, persist.

Early hypotheses and paradigms suggest a homogenous habitat and species-poor ecosystems occurring in the deep sea [24]. Nevertheless, recent research suggests that deep-water ecosystems are, in general, highly heterogeneous and diverse along spatial and temporal axes of variability at different scales [24, 25]. Depth gradients play an important role in governing the structure of deep-sea communities [24, 26], but there is still debate concerning the changes in diversity associated with depth [27]. Contrary to early hypotheses of decreasing diversity with depth, current models of deep-sea diversity assume a unimodal curve with high values of diversity at the continental slope (200–2000 m deep) or continental rise (2000–4000 m) with lower values in deeper habitats [24, 25, 28]. Evidence of diversity patterns as a function of depth, including clues in the Caribbean Basin, are based on the estimation of species ranges [3, 29], which could generate a spurious unimodal model [30]. For the Caribbean Basin, sample-based estimations of diversity applied to deep-water corals have revealed more diversity at the continental slope than on the shelf [31] but species richness in deeper habitats is uncertain. The changes in diversity with depth could also be caused by turnover in species composition, species loss promoted by ecological filters associated with depth, or a combination of both processes [25, 32]. In general, for deep-sea environments, the balance between turnover and nestedness is usually associated with available energy [32], and their identification provides clues about causes of assemblage structure [25]. Although changes in species richness with depth are expected [24, 25], there is no evidence that variation on Caribbean megafauna composition associated with depth is constant at regional scales or among different taxa.

In addition to the general ideas about vertical patterns of deep-sea communities, the existence of regional variation as the depth increases is a point of current debate. For coastal and shelf areas, the Tropical Northwestern Atlantic province is divided into nine ecoregions, five of which are within the Caribbean Sea: Greater Antilles, Eastern Caribbean, Western Caribbean, Southwestern Caribbean and Southern Caribbean [33]. An assessment of various taxa of coastal and shelf benthic megafauna from the Caribbean does not show significant differences in species composition across regions [4, 5], suggesting that ecoregional classification does not apply on this basin. Also an analysis of deep-sea corals suggests a lack of variation in species composition within the Caribbean Sea [29].

Nevertheless, there is evidence suggesting regional variation among the abyssal fauna in the Caribbean (3411–5062 m deep)[34], for deep-water scleractinian corals (> 50 m deep)[3, 35] and in diverse groups of corals on the continental shelf and slope [31]. Additionally, the spatial variation in species composition of deep-sea corals changes between the continental shelf and the slope [31].

**Fig 1:**
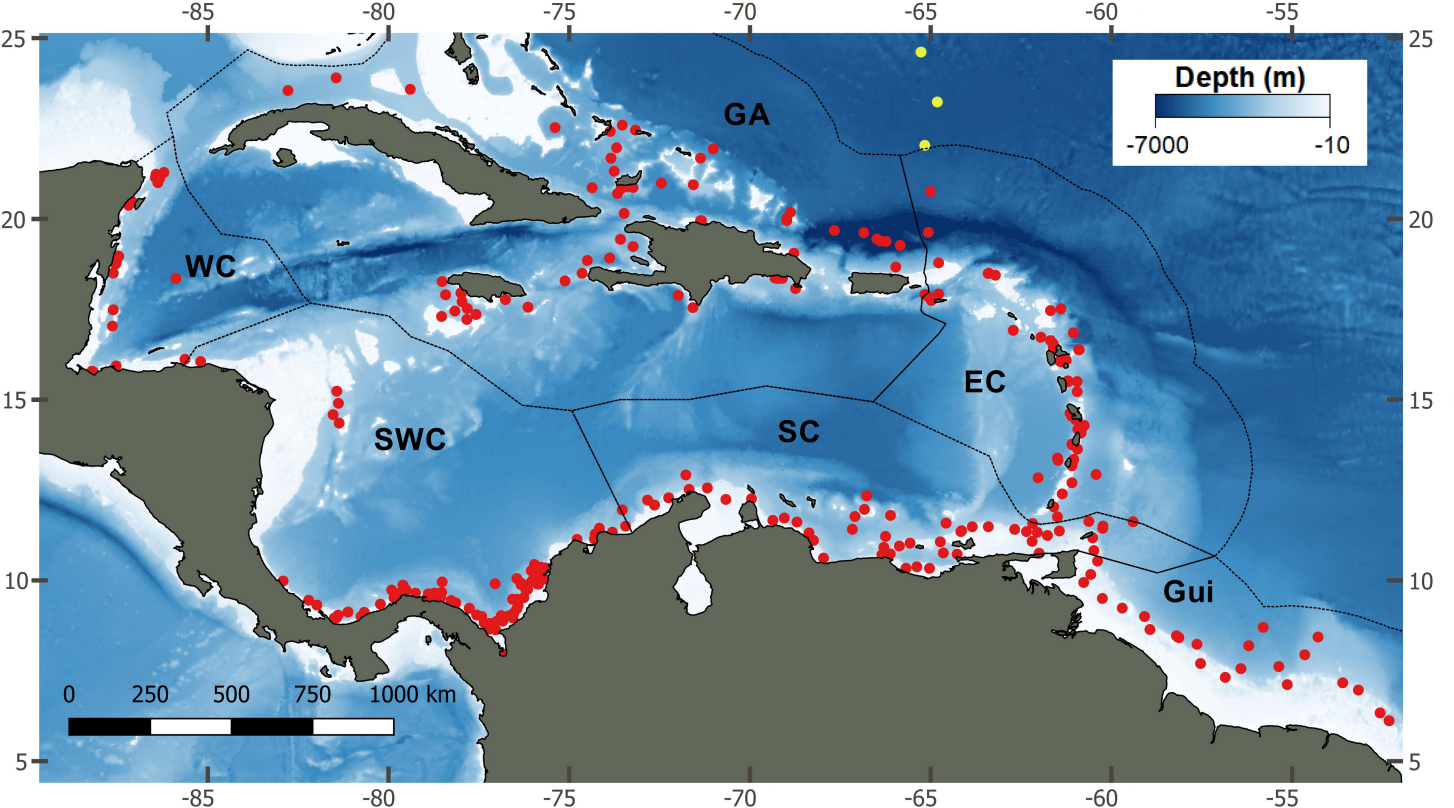
Sampling stations (red dots) along the Caribbean basin and Guiana region. Ecoregions: GA, Greater Antilles; EC, Eastern Caribbean; WC Western Caribbean; SWC, Southwestern Caribbean; SC, Southern Caribbean; Gui, Guiana. Southern locations of the Bahamas were included as Greater Antilles (see methods). Yellow dots are deeper stations not included in ecoregion test, only included in estimation of general diversity below 2000 m depth.

In this study, the patterns of species diversity from the Caribbean deep-sea megafauna were analyzed to test the hypotheses that: 1) megafaunal species diversity changes along Caribbean depth zones, 2) the species composition changes according to ecoregions (for Caribbean ecoregions and the Guianian ecoregion) and, 3) the effect of a depth gradient on megafauna diversity is constant across Caribbean ecoregions and taxa. Data-bases were obtained from seven expeditions performed by the R/V Pillsbury (1966 to 1971), encompassing 310 sites bordering the Caribbean Basin (Fig 1) at depths ranging between 60 and 7500 m [36–42]. The dataset includes records of deep-water corals (Anthozoa: Scleractinia, Alcyonacea, Antipatharia and Hydrozoa: Anthoathecatae), sea stars (Asteroidea), sea urchins (Echinoidea), sea lilies (Crinoidea) and gastropods (Gastropoda). At each site, samples were collected by otter trawls (10-ft or 41 ft) and brought on-board for taxonomic examination. Specimens were identified (in many cases described for first time) by major taxonomic experts and included in many revisions [4, 35, 43]. This broad and standardized sampling effort is ideal to make estimates of species diversity that is comparable between regions, depths and taxa.

## Methods

### Data collection

Data base records were obtained from the National Museum of Natural History, Smithsonian Institution (NMNH-SI) collection database (http://invertebrates.si.edu/collections.htm), and the Marine Invertebrate Museum of the Rosenstiel School of Marine and Atmospheric Sciences (MIM-RSMAS) at the University of Miami where specimens from the R/V Pillsbury expedition were deposited. Station information, collection data and additional information were verified from the NMNH-SI collection database and R/V Pillsbury reports [36–42].

Prior to our analyses, species information were revised and their taxonomy updated according to taxonomic treatises and various online data bases including the World Marine Species Database (www.marinespecies.org/), the Integrated Taxonomic Information Service (www.itis.gov), and Octoclass (http://researcharchive.calacademy.org/research/izg/OCTOCLASS.htm). Information with unresolved inconsistencies were excluded from the dataset. The dataset was sorted as a presence-absence matrix of species per station, thus avoiding duplicate records from collections. Although dataset from the R/V Pillsbury expeditions does not comprise all species and records of Caribbean deep-water species up to date, the dataset could furnish a representative sample for estimate general patterns of diversity. The number of species, the number of records, the station distribution and the fact that collections were performed systematically during short period (1966–1971) by the same research team using one type of gear (trawling) and carefully identified and recorded, provide a reliable information to test diversity patterns hypotheses.

Data were sorted according to major deep-sea zones and ecoregions. The major depth zones include: 1) the shelf, the mesophotic-aphotic realm, 60–200 m depth, close to coastal habitats, and relatively connected with shallow-water systems; 2) the slope, following the shelf, at 200–2000 m depth, aphotic, very close to the coast depending on their profile, and variable coastal influence; 3) the rise, open-sea stations, at 2000–4000 m depth, on the border of the Caribbean continental margin, includes the depth of the Aragonite saturation horizon in the Atlantic (approximately 2500 m deep [44]); 4) the abyssal, internal plains at Caribbean sub-basins, 4000–6000 m deep, and 5) the hadal (> 6000 m, restricted to the Puerto Rico trench)[31, 45]. The biogeographical subdivisions follow Spalding et al. [33] and includes 1) the Greater Antilles (59 Stations), 2) Eastern Caribbean (45 Stations), 3) Western Caribbean (21 Stations), 4) Southern Caribbean (53 Stations), 5) Southwestern Caribbean (79 Stations) and 6) the Guianian Ecoregion (21 Stations). Southern locations of the Bahamian regions were included as part of the Greater Antilles due to a lack of differences in assemblage composition of deep-water corals (following Hernández-Ávila [31]) and on other taxa analyzed in the current study (preliminary analyses) (Fig 1).

### Statistical analyses

#### Species richness

To identify shifts in species richness distributions as a function of depth by ecoregion, the values of richness registered at each sampling site were plotted against depth values. The relationship was fitted using a linear model to detect general trends, as well as with a local polynomial regression to detect potential unimodal relationships. In order to quantitatively compare the species richness at each depth range, sample-based species accumulation curves, based on Chao2 estimations, were plotted using ESTIMATES [46]. Due to sample constraints, records from the continental rise, the abyssal and the hadal depths were pooled into a single depth range, herein called a deeper range. Species richness estimations were extrapolated to 150 samples for each depth range to obtain comparisons under the same sample size. Estimations were performed for each taxon (corals, Asteroidea, Echinoidea, Crinoidea and Gastropoda) and for the merged assemblage. Differences in species richness as a function of depth ranges were tested using *t*-tests based on the parameters obtained by ESTIMATES. In addition, we compared the confidence intervals of each curve [47]. The estimations per taxon were performed considering all previously verified data, including those with null records for a particular taxon, and stations with null records for all taxa, but with verified proper sampling and collections of other megafaunal taxa.

#### Species composition

We assessed the overall multiple-site Sorensen index of dissimilarity for each region, as a proxy of total beta diversity (β_SOR_), as well as the spatial turnover (β_SIM_) and nestedness (β_NES_) components of beta diversity [48] using the *betapart* package implemented in R [49]. These estimators identify whether the change in species composition across sites and depth ranges in each ecoregion is generated by species turnover, species loss, or a combination of both processes. Simultaneously, differences in β-diversity among ecoregions considering two depth ranges (i.e., continental shelf and slope) were tested using multivariate dispersion over the site × site Sorensen dissimilarities matrix [50]. In this case, the null hypothesis is that of homogeneity in the multivariate dispersions among the six ecoregions based on the Sorensen dissimilarity at each of the two depths. The hypotheses of non-differences in species composition between ecoregions, as well as the effects of depth, were tested using Permutational Multivariate Analysis of Variance (PERMANOVA) with depth as a covariate [51]. Additionally, the hypothesis of similar vertical change in species composition across ecoregions was tested considering the interaction ecoregion × depth. The *p-values* for the tests were obtained by 9 999 permutations of residuals under the reduced model. A canonical analysis of principal coordinates (CAP) was used to plot the relationship among change in species composition and depth at each ecoregion [52]. Finally, central tendencies of species composition between depth ranges and ecoregions were represented by bootstrap averages of ecoregions in multidimensional scaling plots (MDS) and their corresponding 95% bootstrap intervals [53].

To identify groups of species that tend to appear together across depth range and ecoregions, a cluster analysis of species was done using the Whittaker’s Index of Association. Only the 60 most frequent species were considered for this analysis. A shade plot (a.k.a. heat map) was used to represent those groups of species according to the correspondent depth range and ecoregion in which they tended to appear [54]. These analyses were done using the matrix display routine in Primer v.7 [55].

## Results

### Vertical and horizontal patterns of species richness

**Fig 2:**
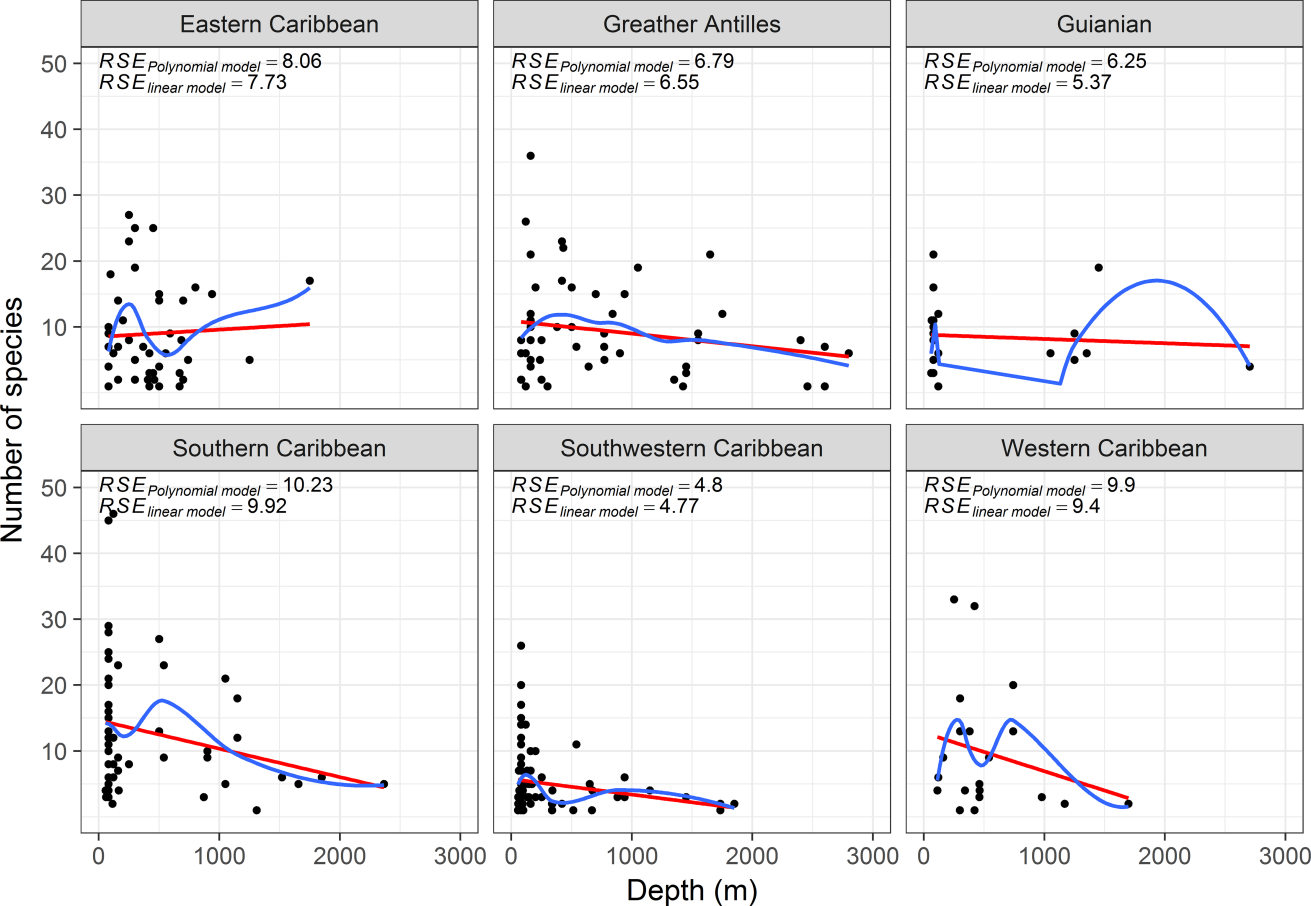
Relationship between numbers of species at each site versus depth and ecoregion. Residual standard errors (RSE) of linear (red) and polynomial models (blue) are shown.

In general, richness tends to decrease linearly with depth. The strength of this relationship was higher in the Southern, Southwest and Western Caribbean (Fig 2) than in the Eastern Caribbean, Guianian and Greater Antilles. However, in all ecoregions, variability in the number of species per site was considerably higher in the continental shelf sites than in deeper sites. The polynomic regression showed no clear pattern of unimodal species richness at continental slopes, but in some cases indicated bimodal species richness (Fig 2), whose peaks varied according to ecoregion. In the Southern, Southwestern and Western Caribbean, bimodal peaks of species richness were observed within the first 1000 m, and in the Eastern Caribbean, Guianian and Greater Antilles, bimodal peaks were observed within the first 500 m as well as the interval 1000–2000 m.

**Fig 3:**
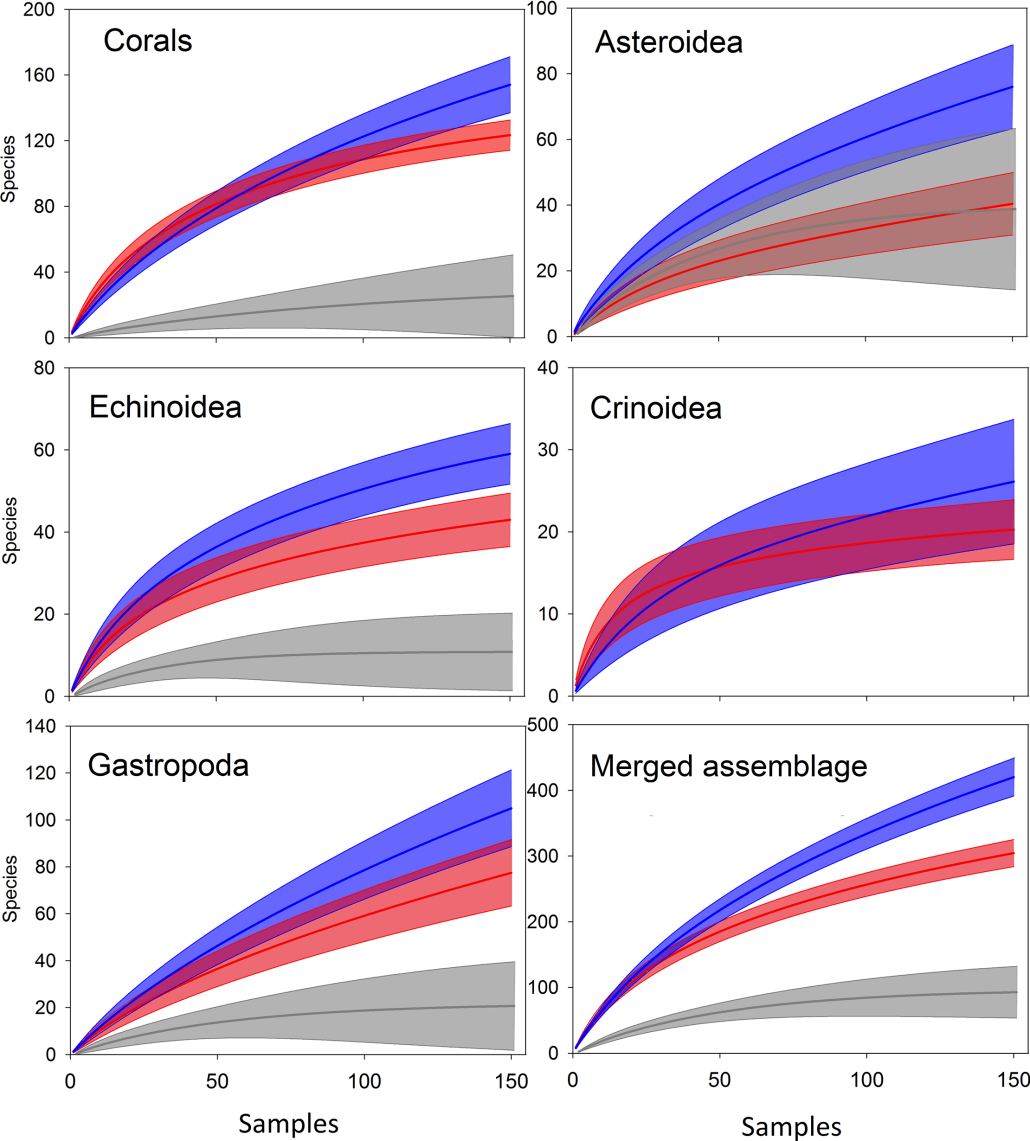
Species accumulation curves and 95% confidence intervals (shaded areas) for the upper continental shelf (red), continental slope (blue), and deeper range (grey) for each taxon and the merged assemblage. All differences between depth ranges were significant in each case (p< 0.05), except between the continental shelf and deeper range for Asteroidea. Crinoidea were not recorded below 2000 m in this data set.

For all taxa, higher species richness was observed on the continental slope than on the continental shelf and in the continental rise-abyssal range (*t*-test, *p* < 0.05, in all cases) (Fig 3). In addition, all taxa but Asteroidea showed lower diversity values for the deeper range (> 2000 m deep) than on the continental shelf (*t*-test, *p* < 0.05). For Crinoidea, few records were observed below a depth of 2000 m. For the total assemblage, the continental slope showed 1.4 times more species (420.12 ± 14.66 spp.) than the upper continental shelf (304.46 ± 10.51 spp.) and 4.5 times more species than the deeper range (> 2000 m deep) (92.83 ± 20.04 spp.). The increment in species number at the continental slope was also observed using both the accumulative species curve and the Chao2 estimator, despite the differences in the approach of the estimators (S1 Fig).

### Vertical and horizontal patterns of species composition

In general, magnitudes of the estimated overall beta diversity (β_SOR_) showed high values in all ecoregion × depth (> 0.95) and for all taxa (> 0.86) (Table 1A and 1C). The partitioning of the Sorensen multi-site index (β_SOR_>> 0.50) [48] indicates that beta diversity across depth and ecoregions is mainly due to species turnover rather than species loss. In addition, there were no differences in multivariate dispersion between ecoregions at the continental shelf (*F* = 2.68, df_d_ = 124, *p* = 0.432). However, on the continental slope, differences in dispersion among ecoregions were detected (PERMDISP, *F* = 8.67, df_d_ = 113, *p* = 0.004), generated mainly by lower dispersions in Guianian and the Southern Caribbean (Table 1B).

**Table 1:** Relative contribution of β-diversity by species turnover (β_SIM_) and species nestedness (β_SNE_) related with Sorensen β-diversity (β_SOR_) [48].

	Eastern Caribbean	Guianian	Greater Antilles	Southern Caribbean	Southwestern Caribbean	Western Caribbean
**A**						
β_SIM_	0.96	0.93	0.97	0.96	0.96	0.94
β_SNE_	0.02	0.03	0.01	0.02	0.02	0.03
β_SOR_	0.98	0.96	0.98	0.98	0.98	0.97
**B**						
MD Cont. Shelf	61.90	61.56	64.88	61.68	64.34	50.34
n Cont. Shelf	12	14	15	35	53	3
MD Cont. Slope	66.25	54.13	66.72	60.86	65.85	66.51
N Cont. Slope	31	5	31	16	26	17
**C**	Cnidaria	Asteroidea	Echinoidea	Crinoidea	Gastropoda	Merged Assemblage
β_SIM_	0.78	0.83	0.79	0.77	0.87	0.85
β_SNE_	0.09	0.08	0.07	0.08	0.06	0.09
β_SOR_	0.87	0.90	0.86	0.86	0.93	0.94

In A, decomposition of Sorensen index by ecoregions. In B, multivariate dispersion (MD) by ecoregions. In C, decomposition of Sorensen index for each particular taxon.

**Fig 4:**
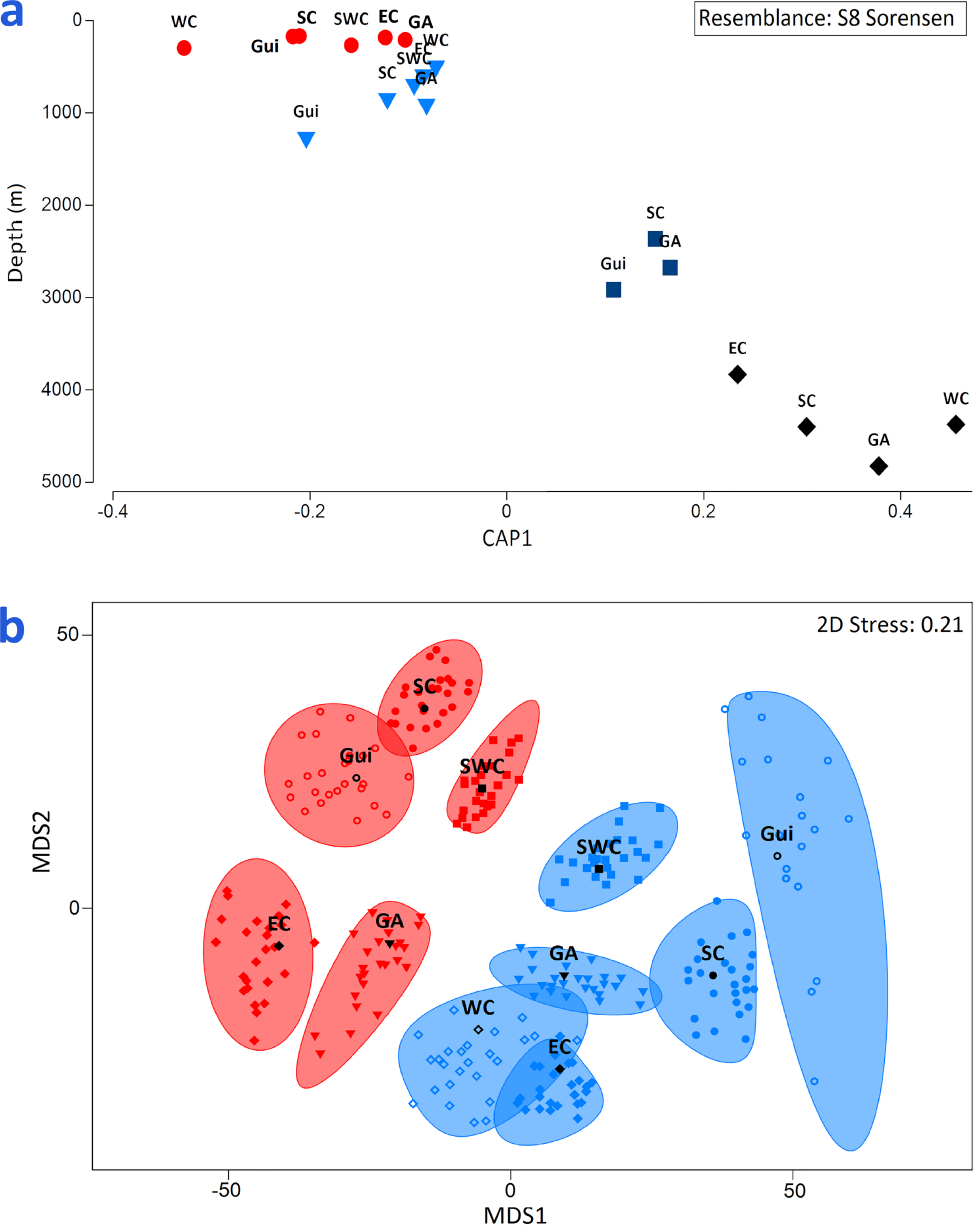
**a. Canonical analysis of principal coordinates (CAP) of ecoregions-depth centroids and mean sampling depth. b. MDS of bootstrap averages of ecoregion-depth centroids for species composition of the merged assemblage for the continental shelf and slope.** a. Deep ranges, symbols: red circles, continental shelf; blue triangles, slope; dark blue squares, bathyal; black rhombus, abyssal. Ecoregions: GA, Greater Antilles; EC, Eastern Caribbean; WC, Western Caribbean; SWC, Southwestern Caribbean; SC, Southern Caribbean; Gui, Guianian. b. Ecoregions at the continental shelf (red) and slope (blue). Ecoregions as 4a. Western Caribbean upper continental shelf was omitted due to insufficient data for bootstrapping. The positions of symbols represent the position of centroids per bootstrap, with dark symbols as averages and shaded areas the 95% confidence intervals.

Potential effects of depth on species composition were not constant across ecoregions, as shown by the significance of the interaction terms depth × ecoregion in most PERMANOVA tests (Table 2). Only the analysis of asteroids indicated that the effect of depth seemed to be constant across ecoregions. In general, depth explained between 7–15% of the total variation, while the effect of ecoregions explained approximately 12–22% of the variation. Fig 4A shows changes in species composition according depths in all ecoregions. Additionally, Fig 4B shows differences between all ecoregions at the continental shelf and slope.

**Table 2:** PERMANOVA tests of species composition (Sorensen) for each major taxon and the merged assemblage.

Taxa/Source	df	SS	MS	Pseudo-F	P(perm)	√CV
**Deep-Water Corals**						
Depth (cov)	1	26549	26549	6.2	**0.0001**	10.69
Ecoregion	5	51224	10245	2.4	**0.0001**	14.00
Depth x Ecoregion	5	48199	9639.7	2.7	**0.0001**	16.09
Residual	183	7.78E+05	4253.9			65.22
Total	194	9.04E+05				
**Asteroidea**						
Depth (cov)	1	17562	17562	4.0	**0.0001**	9.93
Ecoregion	5	38470	7693.9	1.8	**0.0002**	12.70
Depth x Ecoregion	5	24479	4895.8	1.1	0.1644	5.18
Residual	122	5.32E+05	4356.9			66.01
Total	133	6.12E+05				
**Echinoidea**						
Depth (cov)	1	36096	36096	8.8	**0.0001**	13.72
Ecoregion	5	55666	11133	2.7	**0.0001**	16.20
Depth x Ecoregion	5	39201	7840.2	1.9	**0.0001**	12.99
Residual	158	6.47E+05	4094.2			63.99
Total	169	7.78E+05				
**Crinoidea**						
Depth (cov)	1	32333	32333	9.6	**0.0001**	14.81
Ecoregion	5	65557	13111	3.9	**0.0001**	22.14
Depth x Ecoregion	5	43745	8749.1	2.6	**0.0001**	17.45
Residual	120	4.06E+05	3385.4			58.19
Total	131	5.48E+05				
**Gastropoda**						
Depth (cov)	1	21624	21624	5.1	**0.0001**	12.09
Ecoregion	5	43184	8636.7	2.0	**0.0002**	15.71
Depth x Ecoregion	5	28087	5617.5	1.3	**0.0108**	10.57
Residual	107	4.51E+05	4218.7			64.95
Total	118	5.44E+05				
**Merged assemblage**						
Depth (cov)	1	19166	19166	4.26	**0.0001**	7.30
Ecoregion	5	61352	12270	2.72	**0.0001**	13.48
Depth x Ecoregion	5	37566	7513.2	1.67	**0.0001**	9.96
Residual	263	1.18E+06	4503.9			67.11
Total	274	1.30E+06				

Sampling depth is included as covariable (cov). Square root of estimated component of variance (√CV) per source is shown.

A multivariate pairwise *t-*test indicated significant differences in species composition among ecoregions in almost all cases (Table 3). The only exception is the comparison between the Eastern Caribbean and Western Caribbean; however, these two ecoregions are separated by the centre of the Caribbean Basin and by distinct ecoregions (Greater Antilles, Southern and Southwestern Caribbean). Merging of the Eastern Caribbean and Western Caribbean as a single ecoregion is not geographically coherent. Based on these analyses, the separation of all Caribbean and Guianian ecoregions is sustained by changes in the composition of the merged assemblage. Analyses of each taxon separately are consistent with those on the merged assemblage but show more complex patterns where 1) non-differences between the Greater Antilles and Eastern Caribbean in all taxa except for corals were observed; 2) non-differences detected between the Southern Caribbean and Guianian in corals and echinoids; 3) non-differences in sea-star composition between the Greater Antilles and Southwestern Caribbean; 4) lack of differences in gastropod composition in southern ecoregions (Southern, Southwestern Caribbean and Guianian).

**Table 3:** The *p*-values of multivariate pairwise *t-*tests for differences in species composition between ecoregions for the merged assemblage.

Regions	GA	EC	Gui	SC	SWC	WC
GA						
EC	**0.0161**					
Gui	**0.0001**	**0.0001**				
SC	**0.0001**	**0.0001**	**0.0398**			
SWC	**0.0001**	**0.0001**	**0.0010**	**0.0001**		
WC	**0.0072**	0.1490	**0.0003**	**0.0004**	**0.0001**	

Based on 9999 permutations of raw data. Ecoregions: GA, Greater Antilles, EC, Eastern Caribbean, Gui, Guianian, SC, Southern Caribbean, SWC Southwestern Caribbean, WC, Western Caribbean.

### Similarities in species association

**Fig 5:**
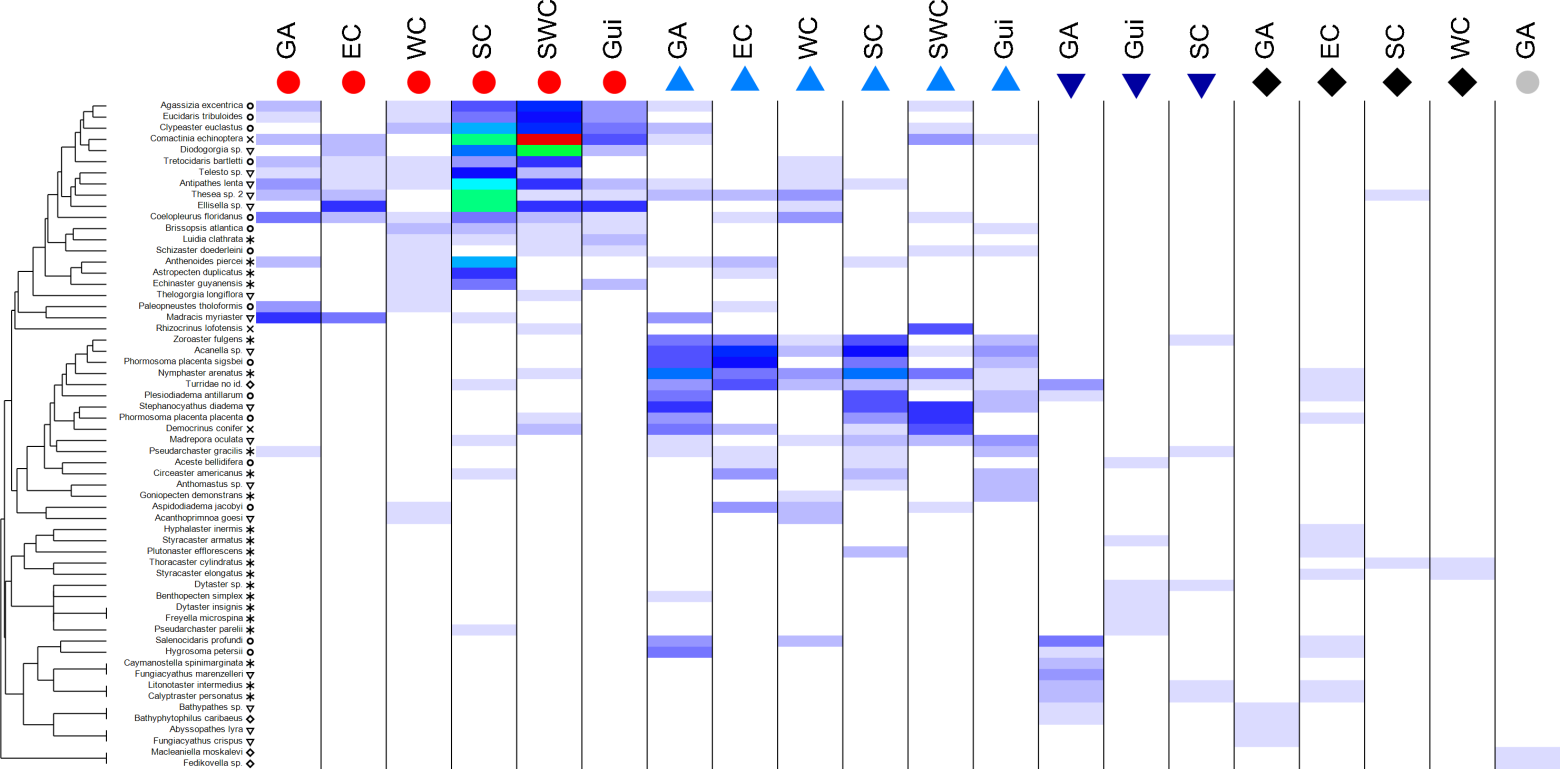
Shade plot and cluster analyses for 60 of the most frequent species based on Whittaker’s Index of Association and its occurrence for each ecoregion-depth. Colors on cells represent the standardized species frequency, during sampling, along ecoregion-depth zone combinations. Top symbols: red circles, continental shelf; blue triangles, slope; dark blue triangles, bathyal; black rhombus, abyssal; gray circle, hadal. Taxa symbols: open triangles, corals; asteriscs, Asteroidea; open circles, Echinoidea; X, Crinoidea; open rhombus, Gastropoda.

Particular patterns for species with a frequent presence at specific depths were observed in almost all ecoregions. This is the case of crinoids *Comactinia echinoptera*, the echinoids *Coelopleurus floridanus*, *Clypeaster euclastus*, *Agassicia excentrica*, and the corals *Telesto* sp., *Antipathes lenta* and *Elisella* sp., for the continental shelf. At the continental slope the corals *Madrepora oculata*, *Stephanocyathus diadema*, *Acanella* sp., the crinoid *Democrinus conifer*, the echinoid *Phormosoma placenta*, and the asteroids *Zoroaster fulgens* and *Nymphaster arenatus* were observed. Species frequently found at the bathyal and abyssal realm included the asteroids *Litonotaster intermedius*, *Calyptraster personatus*, *Dytaster* spp., the echinoid *Salenocidaris profundi*, the coral *Fungicyathus marenzelleri*, and the gastropod *Bathyphytophilus caribaeus* (Fig 5).

## Discussion

### Changes in species richness across ecoregions and depth

The occurrence of high species diversity in deep-water has been a slowly emerging paradigm since the early works of Sanders [56], Dayton and Hessler [57] and Rex [58]. A unimodal distribution of species richness according to depth, with a peak around the continental slope, had been previously proposed for the deep-water fauna [24, 26, 59–61]. However, there are various exceptions to this distribution model [62–64]. In the Caribbean basin, the increase in species richness in coral communities at the continental slope has been previously reported [29, 31]. The probable sources of variation in deep-sea richness patterns according to depth include spatial heterogeneity in substrate characteristics and carbon input [25, 26, 64, 65]. Our findings confirm previous observations on diversity distribution patterns [31] by providing an integrated analysis among different taxa, and show an increment of species diversity from the continental shelf to the slope with a posterior decrease at deeper ranges. A consistent species turnover along depth was observed, in line with the separation of major deep-sea habitats [45], but variation according to depth is different between ecoregions.

Levin et al. [26] underlined that a combination of species distribution as a function of depth and boundary constraints could generate a unimodal model of species without a real association with environmental factors. The results from this study suggest that changes in species composition and richness trends, especially between adjacent depth ranges, are not generated by boundary constraints, but by species turnover. In a model generated merely by boundary constraints, differences in assemblage composition in adjacent ranges could overlap due to the species depth ranges. However, the species depth ranges could have contributed to the model of richness distribution patterns. The wide depth distribution intervals of mesophotic species and the occurrence of endemic deep-water species could contribute to the high diversity observed on the continental slope [29, 31] as well as for the variability in the observed distribution pattern.

The observed decrease in species diversity below the continental slope could be related to deep-sea productivity as a result of particulate organic carbon (POC) flux produced in the photic zone. Carbon supply in deep-sea habitats depends on input from shallow waters except in deep-water chemosynthetic habitats where significant primary productivity from bacteria occurs. POC flux from the ocean upper layer decreases exponentially with depth and only approximately 1% of the primary production is transported below to 1500 m depth [66]. This food deprivation could affect the lower depths by limiting the distribution of some species [67], and hence shape the diversity of the deep ocean [32] at global scale [68]. The decrease of bottom productivity could also have an effect on the reduction of environmental heterogeneity because of the magnitude of differences on the Caribbean primary production, especially in coastal margins [69], which could be mitigated by exponential reduction of POC flux to the depths. However, regional differences in species composition at the continental rise and the abyssal depth are still observed in the Caribbean Basin.

The decrease in diversity below a depth of 2000 m could be related to the depth of the aragonite saturation horizon (ASH, Ω_ARAG_< 1) in the tropical northwestern Atlantic (approximately 2500 m deep) [44]. Low saturation carbonate states are correlated with decreased calcification rates in invertebrates that form shells and carbonate skeletons [70, 71] and seem to limit the depth ranges of most of deep-water corals species [72]. However, the Caribbean bathyal and abyssal zones are not completely deprived of calcified taxa, with persisting species apparently tolerant to Aragonite unsaturated stages [3, 73], as shown in this study. The combined effect of low productivity and Aragonite unsaturated states may represent an ecological filter for species distribution causing species-poor assemblages in deep-sea habitats.

### Variation in species composition across ecoregions and depth

Ecoregional variation on species composition was detected in the studied area. These findings reject previous hypotheses that suggest a homogeneous distribution of the Caribbean deep-sea fauna [4, 5, 29] and support the hypothesis of variation of deep-water Caribbean fauna according to ecoregions and depth. Our results show consistent patterns among taxa suggesting the occurrence of a general trend of heterogeneous distribution, as previously demonstrated for deep-water corals [3, 31]. A marked variation in deep-water assemblages could occur at smaller scales, as observed at the shelf and slope of the Colombian margin [74].

For depth ranges below 2000 m Briggs et al. [34] reported differences in faunal assemblages between three sites at the Venezuelan Basin (at a depth of 3411–5062 m) separated by hundreds of kilometers. Although this variation was detected at the centre of the Venezuelan sub-Basin, it denotes the occurrence of spatial variation of faunal assemblages at continental rise-abyssal depths.

Despite differences in species assemblages among ecoregions, there are different degrees of association between ecoregions, and various species were widespread among samples. Models of deep-water larval dispersal in the Caribbean denote a high potential connectivity between ecoregions and with adjacent areas [75], especially when no major physical barriers are present as in the Caribbean Basin. Ecoregional differences in species assemblages could be driven by ecological more than physical barriers, thus limiting connectivity. Differences in bottom water masses, and changes in environmental conditions such as sediment characteristics and POC flux on the Caribbean seafloor [9, 76], could contribute to the observed differences in species assemblages among ecoregions.

In spite of the general trends described here, it is important to note that our analysis did not include information on particular Caribbean deep-water ecosystems such as hydrothermal vents, submarine volcanoes and cold seeps occurring along the basin. For instance, hydrothermal vent ecosystems have been found at the Cayman Trough, between 2290–4945 m in depth, [21, 77]. Additionally, active venting at a depth of 240–260 m is reported for the Barbados submarine volcano Kick’em Jenny but low hydrothermal fauna is found [22, 78]. Caribbean cold-seeps are found at the El Pilar region (1000–1300 m deep) and the Orinoque sector (1700–2000 m deep), between the mouth of Orinoco River, Trinidad and Tobago, and the Lesser Antilles [19, 79]. Additionally, cold-seep chemosynthetic communities are found at the Colombian margin (La Fuente area, 1000–2500 m deep) [80] and on mud volcanoes at the eastern border of the basin at a depth of 4710–4980 m [81]. In Barbados, at the submarine volcano, Kick’em Jenny, there are occurrences of cold-seep ecosystems associated with volcano debris deposits (1952–2042 m deep), and hydrothermal venting, but few hydrothermal-vent fauna had been found up to date [22, 82]. The cold-seep ecosystems are still largely unexplored but first insights suggest the presence of shared taxa on these ecosystems along the Atlantic Equatorial Belt [17, 83]. Olu et al. [17] proposed that the occurrence of more cold-seep ecosystems spread along the Atlantic equatorial belt (which includes the Caribbean) could serve as stepping-stone habitats for ecosystem connectivity. More exploration of the Caribbean deep-water benthos is necessary to determine the precise distribution of chemosynthetic communities and their diversity patterns.

### Conclusions and general considerations

Patterns of Caribbean deep-water megafauna diversity are similar among the five taxa examined in this study. In general, species diversity increases from the continental shelf to the slope followed by a decrease toward deeper environments. Our findings show that variation in species composition occurs according to ecoregions proposed by Spalding et al. [33] for coastal and shelf systems. The variation in species composition associated with depth also changes according to ecoregions, denoting different patterns of zonation and supporting the hypothesis of ecoregional variation. Patterns of β-diversity among ecoregions and depth range combinations were dominated by species turnover. The heterogeneous distribution of environmental conditions such as bottom topography, POC flux, aragonite saturation, and deep-water masses might act as potential drivers of Caribbean deep-water diversity.

The general distribution trends of species assemblage compositions observed here have important implications in terms of understanding the patterns and drivers of deep-water ecosystems in the Caribbean basin. Historical data, such as those used here, allow observations of the general trends of deep-water megafauna distribution in the Caribbean Basin. Nonetheless, since the data were collected, human threats such as oil exploitation [72], bottom trawling [84, 85] and overfishing could have modified the observed patterns of deep-water diversity. Notwithstanding, managing actions at different scales are important and represent an advance for the conservation of deep-water marine ecosystems in the Caribbean [86]. More regional-scale actions are needed in order to preserve megafaunal diversity in the Caribbean basin, taking into account the diversity patterns of these assemblages.

## Supporting information

S1 Fig Chao2 estimation of species richness and 95% confidence intervals (thin lines) for the upper continental shelf (red) and continental slope (blue) for each taxon and merged assemblage at the Caribbean Basin and Guiana ecoregion. (DOCX)

S1 Table Taxa included in the present study. (XLSX)
