# Supplementary material for: Variation in species diversity of deep-water megafauna assemblages in the Caribbean across depth and ecoregions
Source: PLoS One. 2018 Aug 1;13(8):e0201269. doi: 10.1371/journal.pone.0201269 (PMC6070233; doi:10.1371/journal.pone.0201269)
Supplement: S1 Fig — (DOCX) [file pone.0201269.s001.docx]

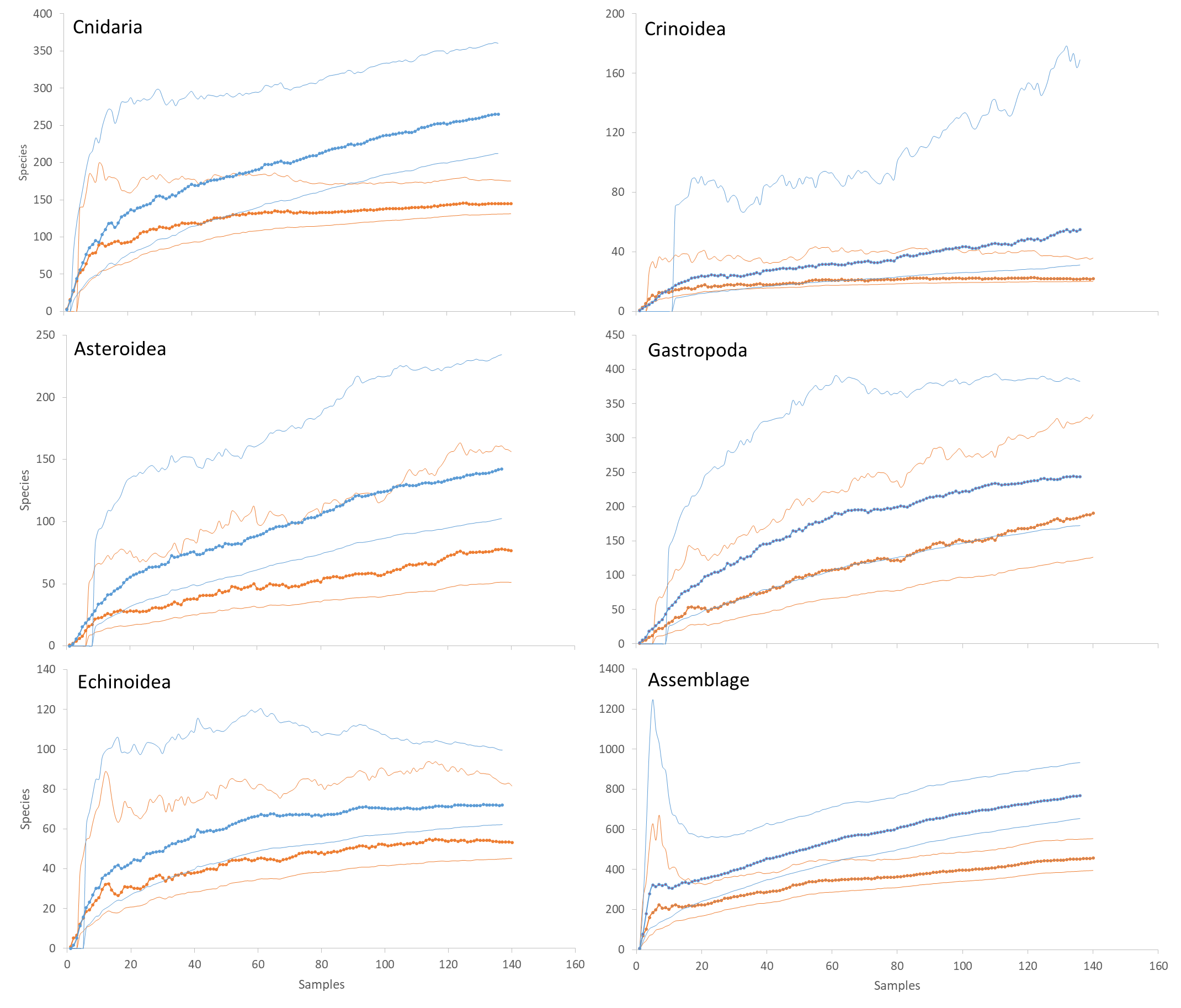


Supplementary material Appendix 1. Fig. A1. Chao2 estimation of species richness and 95% confidence intervals (thin lines) for the upper continental shelf (red) and continental slope (blue) for each taxa and merged assemblage at the Caribbean Basin and Guiana ecoregion. All differences between depth ranges were significant in each case (t-test, p< 0.05).
